# Esters of Quinoxaline 1ˏ4-Di-*N*-oxide with Cytotoxic Activity on Tumor Cell Lines Based on NCI-60 Panel

**Published:** 2017

**Authors:** Gildardo Rivera, Syed Shoaib Ahmad Shah, Daniel Arrieta-Baez, Isidro Palos, Antonio Mongue, Luvia Enid Sánchez-Torres

**Affiliations:** a *Centro de Biotecnología Genómica, Instituto Politécnico Nacional, Reynosa, México. *; b *Department of Chemistry, Quaid-i-Azam University, Islamabad, Pakistan. *; c *Centro de Nanociencias y Micro y Nanotecnología, Instituto Politécnico Nacional, Ciudad de México, México. *; d *Departamento de Química Aplicada, Universidad Autónoma de Tamaulipas, Reynosa, México. *; e *Neglected Diseases Section, Drug R& D Unit, Center for Applied Pharmacobiology Research, University of Navarra, Pamplona, España. *; f *Departamento de Inmunología, Escuela Nacional de Ciencias Biológicas, Instituto Politécnico Nacional, Ciudad de México, México.*

**Keywords:** Antitumor, Cancer, Drugs, Esters, Quinoxaline 1, 4-di-*N*-oxide

## Abstract

Quinoxalines display diverse and interesting pharmacological activities as antibacterial, antiviral, antiparasitic and anticancer agents. Particularly, their 1ˏ4-di-*N*-oxide derivatives have proved to be cytotoxic agents that are active under hypoxic conditions as that of solid tumours. A new series of quinoxaline 1ˏ4-di-*N*-oxide substitutes at 7-position with esters group were synthetized and characterized by infrared (IR), proton nuclear magnetic resonance (^1^H-NMR), spectroscopy, and elemental analysis. Seventeen derivatives (M1-M3, E1-E8, P1-P3 and DR1-DR3) were selected and evaluated for antitumor activities using the NCI-60 human tumor cell lines screen. Results showed that E7, P3 and E6 were the most active compounds against the cell lines tested. Substitutions at 7-position with esters group not necessarily affect the biological activity, but the nature of the esters group could exert an influence on the selectivity. Additionally, substitutions at 2-position influenced the cytotoxic activity of the compounds.

## Introduction

Cancer remains a major cause of death affecting millions of people and is caused by the growth and spreading of abnormal cells in an uncontrolled manner. According to the World Health Organization (WHO) cancer is a leading cause of death worldwide that accounted for 8.2 million deaths in 2012. In addition to the extensive use of irradiation and surgical treatment, chemotherapy still plays an important role for the treatment of cancer (1). In this context, the search for novel chemotherapeutic agents is an interesting and continually evolving field of cancer research. Although major advances have been made in the chemotherapeutic management of patients with certain types of cancer, the continued commitment to the laborious task of discovering new anticancer agents remains critically important. 

In the course of identifying various chemical substances which may serve as leads for designing novel antitumor agents, many classes of organic compounds have been tested, with special attention being paid to nitrogen heterocycles, five- and six-membered rings. Quinoxaline (benzopyrazine) is a heterocyclic compound containing fused benzene and pyrazine rings and it is an isomer of quinazoline. Quinoxalines display diverse and interesting pharmacological activities as antibacterial, antiviral, antiparasitic and anticancer agents (2-8). Particularly, their 1ˏ4-di-*N*-oxide have proved to be cytotoxic agents that are active under hypoxic conditions as that of solid tumours (6, 9-12). Additionally, this kind of derivatives has shown anti-inflammatory and anti-oxidant activities both interesting properties against cancer cells (3, 13). The identification of drugs with selective toxicity toward hypoxic cells is an important objective in anticancer chemotherapy and quinoxaline 1ˏ4-di-*N*-oxides could provide useful hypoxia-selective therapeutic agents. 

In the present work, we are interested in quinoxaline 1, 4-di-*N*-oxide derivatives which have been identified as a new class of cancer chemotherapeutic agents with significant therapeutic efficacy against solid tumours (11-12). It is reported that the antitumor activity can be increased by introducing electron-withdrawing groups in one of both positions 6 and 7 on the quinoxaline ring and in the unsubstituted analogues (14). Therefore, a new series of quinoxaline 1ˏ4-di-*N*-oxide substitutes at 7-position with esters group were synthetized and evaluated for antitumor activities using the NCI-60 human tumor cell lines screen (15).

## Experimental

*Chemical compounds*

The Beirut reaction was the general procedure used for synthesis of quinoxaline 1ˏ4-di-*N*-oxide derivatives, as described in previous report (16, 17). Quinoxaline 1ˏ4-di-*N*-oxide derivatives (M1-M3, E1-E8 and P1-P3) synthesis was achieved by the reaction of the corresponding diketone derivative (10.6 mmol) with the appropriate benzofuroxane *N*-oxide (2.4 mmol) in dry chloroform (35 mL). Triethylamine (TEA) was added (1 mL) and the reaction mixture was stirred at room temperature for 3–7 days. After evaporation to dryness at low pressure, crude solid or brown oil was obtained. This was then precipitated and washed by adding diethyl ether, affording the target compound. The residue was purified by column chromatography on silica gel, when necessary using dichloromethane: methanol (95:5). Quinoxaline di-reduced (DR1-DR3) was achieved by the reaction of the corresponding quinoxaline 1ˏ4-di-*N*-oxide with sodium dithionite (18). All compounds were characterized by infrared (IR), proton nuclear magnetic resonance (^1^H-NMR) spectroscopy, and elemental analysis.

*Methyl quinoxaline-7-carboxylate* *1ˏ4* *-di-N-oxide derivatives*

*Methyl2-amide-3-methylquinoxaline-7-carboxylate 1ˏ4-di-N-oxide *(M1)

This compound was obtained in 10% yield from methyl benzofuroxane-5-carboxylate *N*-oxide and acetoacetamide. IR (KBr): 3310 (NH), 2992 (ArC-H), 1702 (C=O), 1331 (*N*-oxide) cm^-1^. ^1^H NMR (400 MHz, DMSO-*d*_6_) δ ppm: 2.48 (s, 3H, CH_3_), 3.98 (s, 3H, CH_3_OOC), 8.25 (s, 2H, NH_2_), 8.37-8.42 (m, 1H, H5), 8.57-8.62 (m, 1H, H6), 8.94 (s, 1H, H8). Calculated analysis for C_12_H_11_N_3_O_5_: C, 51.98; H, 3.97; N, 15.16. Found: C, 51.72; H, 3.63; N, 14.86.

*Dimethyl3-methylquinoxaline-2, 7-dicarboxylate 1, 4-di-N-oxide *(M2)

This compound was obtained in 12% yield from methyl benzofuroxane-5-carboxylate* N*-oxide and methyl acetoacetate. IR (KBr): 1715.30 (C=O), 1333.57 (*N*-oxide) cm^-1^. ^1^H NMR (400 MHz, DMSO-*d*_6_) δ ppm: 2.44 (s, 3H, CH_3_), 3.97 (s, 3H, COOCH_3_), 4.03 (s, 3H, CH_3_OOC), 8.38 (d, J= 8.93 Hz, 1H, H5), 8.56 (d, J= 8.89 Hz, 1H, H6), 8.85(s, 1H, H8). Calculated analysis for C_13_H_12_N_2_O_6_: C, 53.43; H, 4.14; N, 9.59. Found: C, 53.29; H, 3.97; N, 9.45. 

*Methyl 2-acetyl-3 trifluoromethylquinoxaline-7-carboxylate* *1ˏ4-* *di-N-oxide *(M3) 

This compound was obtained in 17.7% yield from methyl benzofuroxane-5-carboxylate *N*-oxide and 1,1, 1-trifluoro-2,4-pentanedione. IR (KBr): 2962 (Ar C-H), 1732 (C=O), 1337 (*N* - oxide), 1236 and 1173 (Ar-CF_3_) cm^-1^.^ 1^H NMR (400 MHz, DMSO-*d*_6_) ppm: 2.62 (s, 3H, COCH_3_), 3.99 (s, 3H, CH_3_OOC), 8.51 (d, J= 8.95, Hz, 1H, H5), 8.58 (d, J= 8.94, 1H, H6), 8.92 (s, 1H, H8). Calculated analysis for C_13_H_9_F_3_N_2_O_5_: C, 47.28; H, 2.75; N, 8.48. Found: C, 47.01; H, 2.56; N 8.45.

*Ethyl quinoxaline-7-carboxylate* *1ˏ4-* *di-N-oxide derivatives*

*Ethyl2-acetyl-3-methyl-quinoxaline-7-carboxylate* *1ˏ4-* *di-N-oxide *(E1)

This compound was obtained in 28% yield from ethylbenzofuroxane-5-carboxylate *N*-oxide and 2, 4-pentanedione. IR (KBr): 2965 (ArC-H), 1722 (C=O), 1335 (*N*-oxide) cm^-1^. ^1^H NMR (400 MHz, DMSO-*d*_6_) δ ppm: 1.40 (t, J= 7.10 Hz, 3H, CH_3_CH_2_OOC), 2.38 (s, 3H, CH_3_), 2.66 (s, 3H, COCH_3_), 4.4 (q, J= 7.09 Hz, 2H, CH_3_CH_2_OOC), 8.37 (d, J= 8.93 Hz, 1H, H5), 8.5 (d, J= 8.94 Hz, 1H, H6), 8.92 (s, 1H, H8). Calculated analysis for C_14_H_14_N_2_O_5_: C, 57.93; H, 4.82; N, 9.65. Found: C, 57.68; H, 4.51; N, 9.18.

*Ethyl2-benzoyl-3-methylquinoxaline-7-carboxylate 1* *ˏ* *4-di-N-oxide *(E2)

This compound was obtained in 12.3% yield from ethyl benzofuroxane-5-carboxylate *N*-oxide and 1-phenyl-1, 3-butanedione. IR (KBr): 2979 (ArC-H), 1720 and 1684 (C=O), 1331 (*N*-oxide) cm^-1^. ^1^H NMR (400 MHz, DMSO-*d*_6_) δ ppm: 1.41 (t, J= 7.11 Hz, 3H, CH_3_CH_2_OOC), 2.32 (s, 3H, CH_3_), 4.46 (q, J_1_= 7.10 Hz, J_2_= 7.13 Hz, 2H, CH_3_CH_2_OOC), 7.6 (t, J= 7.8 Hz, 2H, H3 and H5, C_6_H_5_), 7.79 (t, J= 7.43 Hz, 1H, H4, C_6_H_5_), 8.1 (d, J= 7.34 Hz, 2H, H2 and H6, C_6_H_5_), 8.38 (d, J= 8.9 Hz, 1H, H5), 8.5 (d, J= 8.94 Hz, 1H, H6), 8.99 (s, 1H, H8). Calculated analysis for C_19_H_16_N_2_O_5_: C, 64.77; H, 4.58; N, 7.95. Found: C, 64.58; H, 4.32; N, 7.67.

*Ethyl 2-phenylamide-3-methylquinoxaline-7-carboxylate 1* *ˏ* *4-di-N-oxide *(E3)

This compound was obtained in 22.4% yield from ethyl benzofuroxane-5-carboxylate *N*-oxide and 3-oxo-*N*-phenylbutanamide. IR (KBr): 2981 (ArC-H), 1714 and 1661 (C=O), 1369 (*N*-oxide) cm^-1^. ^1^H NMR (400 MHz, DMSO-*d*_6_) δ ppm: 1.36 (m, 3H, CH_3_CH_2_OOC), 2.51 (s, 1H, CH_3_), 4.39-4.47 (q, J_1_= 7.10 Hz, J_2_= 7.22 Hz, 2H, CH_3_CH_2_O), 7.20 (t, J= 7.34 Hz, 1H, H4-NHC_6_H_5_), 7.41 (t, J= 7.16 Hz, 2H, H3 and H5, NHC_6_H_5_), 7.65 (d, J= 7.84 Hz, H2 and H6, NHC_6_H_5_), 8.39 (d, J= 8.98 Hz, 1H, H5), 8.59 (d, J= 8.97 Hz, 1H, H6), 8.96 (s, 1H, H8), 11.10 (s, 1H, NH). Calculated analysis for C_19_H_17_N_3_O_5_: C, 62.12; H, 4.66; N, 11.44. Found: C. 61.98; H, 4.49; N, 11.23.

*Ethylmethyl-3-methyl-quinoxaline-2 ,7-dicarboxilate 1ˏ4-di-N-óxide *(E4)

This compound was obtained in 10% yieldfromethylbenzofuroxane-5-carboxylate *N*-oxide and methyl acetoacetate. IR (KBr): 1715.30 and 1726.88 (C=O), 1327.65 (*N*-oxide) cm^-1^. ^1^H NMR (400 MHz, DMSO-*d*_6_) δ ppm: 1.35 (s, 3H, CH_3_CH_2_OOC), 2.44 (s, 3H, CH_3_), 4.03 (s, 3H, COOCH_3_), 4.48 (q, J_1_= 7.08 Hz, J_2_= 7.07 Hz, 2H, CH_3_CH_2_OOC), 8.39 (d, J= 8.99 Hz, 1H, H5), 8.56 (d, J= 8.99 Hz, 1H, H6), 8.84 (s, 1H, H8). Calculated analysis for C_14_H_14_N_2_O_6_: C, 54.90; H, 4.61; N, 9.15. Found: C, 54.76; H, 4.27; N, 9.13

*Ethyl 2-acetyl-3-trifluoromethylquinoxaline-7-carboxylate 1* *ˏ* *4-di-N-oxide* (E5)

This compound was obtained in 11.9% yield from ethyl benzofuroxane-5-carboxylate *N*-oxide and 1,1, 1-trifluoro-2,4-pentanedione. IR (KBr): 2978 (ArC-H), 1746 (C=O), 1355 (*N*-oxide), 1271 and 1158 (Ar-CF_3_) cm^-1^. ^1^H NMR (400 MHz, DMSO-*d*_6_) ppm: 1.40 (t, J= 7.11 Hz, 3H, CH_3_CH_2_OOC), 2.62 (s, 3H, COCH_3_), 4.45 (q, J_1_= 7.12 Hz, J_2_= 7.13 Hz, 2H, CH_3_CH_2_OOC), 8.52 (d, J= 8.95 Hz, 1H, H5), 8.58 (d, J= 8.96 Hz, 1H, H6), 8.92 (s, 1H, H8). Calculated analysis for C_14_H_11_ F_3_N_2_O_5_: C, 48.85; H, 3.22; N, 8.14. Found: C, 48.58; H, 3.15; N, 8.23.

*Ethyl2-(thiophene-2-carbonyl)-3-trifluoromethylquinoxaline-7-carboxylate 1* *ˏ* *4-di-N-oxide *(E6) 

This compound was obtained in 23.2% yield from ethyl benzofuroxane-5-carboxylate *N*-oxide and 4,4,4-trifluoro-1-(2-thienyl)-1,3-butanedione. IR (KBr): 2987 (ArC-H), 1726 and 1665 (C=O), 1336 (*N*-oxide), 1286 and 1161 (Ar-CF_3_) cm^-1^. ^1^H NMR (400 MHz, DMSO-*d*_6_) δ ppm: 1.41 (t, J= 7.1 Hz, 3H, CH_3_CH_2_OOC), 4.47 (q, J_1_= 7.07 Hz, J_2_= 7.12 Hz, 2H, CH_3_CH_2_OOC), 7.32 (d, J= 4.7 Hz, 1H, H4, C_4_H_3_S), 8.24 (d, J= 4.59 Hz, H5, C_4_H_3_S), 8.3 (d, J= 4.8 Hz, H3, C_4_H_3_S), 8.51 (d, J= 8.95 Hz, 1H, H5), 8.56 (d, J= 8.96 Hz, 1H, H6), 8.96 (s, 1H, H8). Calculated analysis for C_17_H_11_F_3_N_2_O_5_S: C, 49.52; H, 2.69; N, 6.79. Found: C, 49.36; H, 2.45; N, 6.43.

*Ethyl2-(naphthyl-2-carbonyl)-3-trifluoromethylquinoxaline-7-carboxylate1ˏ4-di-N-oxide *(E7)

This compound was obtained in 11.3% yield from ethyl benzofuroxane-5-carboxylate* N*-oxide and 4, 4, 4-trifluoromethyl-1-(2-naphthyl)-1, 3-butanedione. IR (KBr): 2979 (ArC-H), 1725 and 1687 (C=O), 1349 (*N*-óxido), 1285 and 1174 (Ar-CF_3_) cm^-1^. ^1^H NMR (400 MHz, DMSO-*d*_6_) ppm: 1.42 (t, J= 7.10 Hz, 3H, CH_3_CH_2_OOC), 4.48 (q, J_1_= 7.08 Hz, J_2_= 7.11 Hz, 2H, CH_3_CH_2_OOC), 7.65 (t, J= 7.5 Hz, 1H, H3, C_10_H_7_), 7.75 (t, J= 7.2 Hz, 1H, H6, C_10_H_7_), 8.01 (d, J= 8.12 Hz, 1H, H7, C_10_H_7_), 8.07 (d, J= 8.14 Hz, 1H, H5, C_10_H_7_), 8.14 (s, 2H, H2, and H4 C_10_H_7_), 8.52-8.58 (m, 2H, H5 and H6), 8.88 (s, 1H, H8), 9.02 (s, 1H, H8, C_10_H_7_). Calculated analysis for C_23_H_15_F_3_N_2_O_5_: C, 60.53; H, 3.31; N, 6.14. Found: C, 60.23; H, 3.15; N, 5.89.

*Ethyl 2-phenylamide-3-phenylquinoxaline-7-carboxylate 1, 4-di-N-oxide *(E8)

This compound was obtained in 31.0% yield from ethyl benzofuroxane-5-carboxylate *N*-oxide and 3-oxo-*N , *3-diphenylpropanamide. IR (KBr): 3110 (N-H), 2950 (ArC-H), 1718 and 1694 (C=O), 1334 (*N*-oxide) cm^-1^. ^1^H NMR (400 MHz, DMSO-*d*_6_) δ ppm: 1.41 (t, J= 7.11Hz, 3H, CH_3_CH_2_OOC), 4.45 (q, H= 7.12 Hz, 2H, CH_3_CH_2_OOC), 7.11 (t, J= 7.25 Hz, 1H, H4, NHC_6_H_5_), 7.31 (t, J= 7.88 Hz, 2H, H3 and H5, C_6_H_5_), 7.38 (d, J = 8.23 Hz, 2H, H3 and H5, NHC_6_H_5_), 7.48-7.50 (m, 3H, C_6_H_5_), 7.61-7.63 (m, 2H, H2 and H6, NHC_6_H_5_), 8.49 (d, J= 8.96 Hz, H5), 8.69 (d, J= 8.94 Hz, 1H, H6), 9.02 (s, 1H, H8), 10.86 (s, 1H, NH). Calculated analysis for C_24_H_19_N_3_O_5_: C, 67.13; H, 4.42; N, 9.79. Found: C, 66.95; H, 4.21; N, 9.52.

*n-propil quinoxaline-7-carboxylate 1ˏ4-di-N-oxide derivatives*

*n-propyl 2-benzoyl-3-methylquinoxaline-7-carboxylate 1ˏ4-di-N-oxide* (P1)

This compound was obtained in 8.3% yield from n-propyl benzofuroxane-5-carboxylate *N*-oxide and 1-phenyl-1, 3-butanedione. IR (KBr): 2978 (ArC-H), 1718 and 1685 (C=O), 1332 (*N*-oxide) cm^-1^. ^1^H NMR (400 MHz, DMSO-*d*_6_) δ ppm: 1.02 (t, J= 7.38 Hz, 3H, CH_3_CH_2_CH_2_OOC), 1.81 (q, J_1_= 6.76 and J_2_= 14.12 Hz, 2H, CH_3_CH_2_CH_2_OOC), 4.39 (t, J= 6.62 Hz, 2H, CH_3_CH_2_CH_2_OOC), 7.61 (t, J= 7.82 Hz, 2H, H3 and H5, C_6_H_5_), 7.80 (t, J= 7.40 Hz, 1H, H4, C_6_H_5_), 8.12 (d, J= 7.30 Hz, 2H, H2 and H6, C_6_H_5_), 8.39 (d, J= 8.9 Hz, 1H, H5), 8.51 (d, J= 8.90 Hz, 1H, H6), 8.98 (s, 1H, H8). Calculated analysis for C_20_H_18_N_2_O_5_: C, 65.57; H, 4.91; N, 7.65. Found: C, 65.38; H, 4.63; N, 7.37.

*n-propyl2-phenylamide-3methylquinoxaline-7-carboxylate 1ˏ4-di-N-oxide* (P2)

This compound was obtained in 10.4% yield from n-propyl benzofuroxane-5-carboxylate *N*-oxide and 3-oxo-*N*-phenylbutanamide. IR (KBr): 2982 (ArC-H), 1716 and 1672 (C=O), 1332 (*N*-oxide), cm^-1^. ^1^H NMR (400 MHz, DMSO-*d*_6_) δ ppm: 1.03 (t, J= 7.40 Hz, 3H, CH_3_CH_2_CH_2_OOC), 1.82 (q, J_1_= 6.77 and J_2_= 14.11 Hz, 2H, CH_3_CH_2_CH_2_OOC), 4.38 (t, J= 6.60 Hz, 2H, CH_3_CH_2_CH_2_OOC), 7.21 (t, J= 7.32 Hz, 1H, H4-NHC_6_H_5_), 7.42 (t, J= 7.20 Hz, 2H, H3 and H5, NHC_6_H_5_), 7.64 (d, J= 7.80 Hz, H2 and H6, NHC_6_H_5_), 8.38 (d, J= 8.90 Hz, 1H, H5), 8.58 (d, J= 8.90 Hz, 1H, H6), 8.95 (s, 1H, H8), 11.12 (s, 1H, NH). Calculated analysis for C_20_H_19_N_3_O_5_: C, 62.99; H, 4.98; N, 11.02. Found: C. 62.58; H, 4.61; N, 10.87.

*n-propyl2-benzoyl-3-trifluoromethylquinoxaline-7-carboxylate 1ˏ4-di-N-oxide* (P3)

This compound was obtained in 5.9% yield from n-propyl benzofuroxane-5-carboxylate *N*-oxide and 4, 4,4-trifluoro-1-phenyl-1,3-butanedione. IR (KBr): 2982 (Ar C-H), 1728 and 1688 (C=O), 1336 (*N*-oxide), 1254 and 1166 (Ar-CF_3_) cm^-1^. ^1^H NMR (400 MHz, DMSO-*d*_6_) δ ppm: 1.02 (t, J= 7.40 Hz, 3H, CH_3_CH_2_CH_2_OOC), 1.82 (q, J_1_= 6.77 and J_2_= 14.11 Hz, 2H, CH_3_CH_2_CH_2_OOC), 4.39 (t, J= 6.58 Hz, 2H, CH_3_CH_2_CH_2_OOC), 7.62 (t, J= 7.84 Hz, 2H, H3 and H5, C_6_H_5_), 7.79 (t, J= 7.43 Hz, 1H, H4, C_6_H_5_), 8.15 (d, J= 7.26 Hz, 2H, H2 and H6, C_6_H_5_), 8.52-8.54 (m, 2H, H5 and H6), 8.98 (s, 1H, H8). Calculated analysis for C_20_H_15_F_3_N_2_O_5_: C, 57.14; H, 3.57; N, 6.66. Found: C, 56.91; H, 3.39; N, 6.37.

*Quinoxaline-7-carboxylate derivatives*

*Methyl2-amide-3-methylquinoxaline-7-carboxylate *(DR1)

This compound was obtained in 4% yield from methyl 2 -amide-3-methylquinoxaline-7-carboxylate 1, 4-di-*N*-oxide by di-reduced using sodium dithionite. IR (KBr): 3312 (NH), 2994 (ArC-H), 1702 (C=O) cm^-1^. ^1^H NMR (400 MHz, DMSO-*d*_6_) δ ppm: 2.48 (s, 3H, CH_3_), 3.98 (s, 3H, CH_3_OOC), 8.25 (s, 2H, NH_2_), 8.37-8.42 (m, 1H, H5), 8.57-8.62 (m, 1H, H6), 8.94 (s, 1H, H8). Calculated analysis for C_12_H_11_N_3_O_3_: C, 58.77; H, 4.48; N, 17.14. Found: C, 58.81; H, 4.65; N, 17.23.

*Methyl 2-phenylamide-3-methylquinoxaline-7-carboxylate* (DR2)

This compound was obtained in 6.5% yield from methyl 2-phenylamide-3-methylquinoxaline-7-carboxylate 1, 4-di-*N*-oxide by di-reduced using sodium dithionite. IR (KBr): 3081 (N-H), 2949 (ArC-H), 1722 and 1684 (C=O) cm^-1^. ^1^H NMR (400 MHz, DMSO-*d*_6_) δ ppm: 2.52 (s, 3H, CH_3_), 3.99 (s, 3H, CH_3_OOC), 7.20 (t, J= 7.38 Hz, 1H, H4, NHC_6_H_5_), 7.42 (t, J= 7.78 Hz, 2H, H3 and H5, NHC_6_H_5_), 7.67 (d, J = 7.92 Hz, H2 and H6, NHC_6_H_5_), 8.41 (d, J= 8.84 Hz, H5), 8.62 (d, J= 8.93 Hz, 1H, H6), 9.0 (s, 1H, H8), 11.01 (s, 1H, NH). Calculated analysis for C_18_H_15_N_3_O_3_: C, 67.28; H, 4.67; N, 13.08. Found: C, 67.15; H, 4.23; N, 12.86.

*Ethyl2-(thiophene-2-carbonyl)-3-trifluoromethylquinoxaline-7-carboxylate *(DR3)

This compound was obtained in 2.3% yield from ethyl 2-(thiophene-2-carbonyl)-3-trifluoromethylquinoxaline-7-carboxylate 1, 4-di-*N*-oxide by di-reduced using sodium dithionite. IR (KBr): 2991 (ArC-H), 1722 and 1672 (C=O), 1284 and 1162 (Ar-CF_3_) cm^-1^. ^1^H NMR (400 MHz, DMSO-*d*_6_) ppm: 1.41 (t, J= 7.1 Hz, 3H, CH_3_CH_2_OOC), 4.47 (q, J_1_= 7.07 Hz, J_2_= 7.12 Hz, 2H, CH_3_CH_2_OOC), 7.32 (d, J= 4.7 Hz, 1H, H4, C_4_H_3_S), 8.24 (d, J= 4.59 Hz, H5, C_4_H_3_S), 8.3 (d, J= 4.8 Hz, H3, C_4_H_3_S), 8.51 (d, J= 8.95 Hz, 1H, H5), 8.56 (d, J= 8.96 Hz, 1H, H6), 8.96 (s, 1H, H8). Calculated analysis for C_17_H_11_F_3_N_2_O_3_S: C, 53.61; H, 2.89; N, 7.35. Found: C, 53.46; H, 2.75; N, 

7.23. Compounds derivatives from quinoxaline-7-carboxylate 1ˏ4-di-*N*-oxide (M1-M3, E1-E8, and P1-P3) and quinoxaline di-reduced derivatives (DR1-DR3) are shown in Table 1.

*Biological assays*

The National Cancer Institute (NCI) of the United States of America selected the 17 compounds cited above to be evaluated for their *in vitro *antitumor activity. Initially, all compounds were tested at a single high dose (10^-5^ M) according to NCI-60 human tumor cell line screen. The human tumor cell lines are grown in RPMI 1640 medium (5% fetal bovine serum and 2 mM L-glutamine). Cells are seeded into 96 well microtiter plates in 100 μL (5,000 to 40,000 cells/well). After, the microtiter plates are incubated at 37 °C, 5% CO_2_, 95% air and 100% relative humidity for 24 h. Aliquots of 100 μL experimental drugs are added to the appropriate microtiter wells already containing 100 μL of medium, resulting in the required final drug concentration. After, the plates are incubated for an additional 48 h at 37 °C, 5% CO_2_, 95% air, and 100% relative humidity. For adherent cells, the assay is terminated by the addition of cold TCA. Cells are fixed in situ by the addition of 50 μL of cold 50% (w/v) TCA (final concentration, 10% TCA) and incubated for 60 minutes at 4 °C. The supernatant is discarded, and the plates are washed five times with water and air dried. Sulforhodamine B (SRB) solution (100 μL) at 0.4% (w/v) in 1% acetic acid is added to each well, and plates are incubated for 10 minutes at room temperature. After staining, unbound dye is removed by washing five times with 1% acetic acid and the plates are air dried. Bound stain is subsequently solubilized with 10 mM trizma base, and the absorbance is read on an automated plate reader at a wavelength of 515 nm. After, only compounds M3, E5, E6, E7 and P3 were evaluated against the NCI-60 cell panel at five concentrations (0.01, 0.1, 1, 10 and 100 µm) and Growth Inhibition of 50% (GI50) was calculated. Total Growth Inhibition (TGI) and the Lethal Concentration 50 (LC50) were calculated for each cell line according to NCI methodology (15). Data are express in Molar concentration. The GI50 value corresponds to the concentration of the compound causing 50% decrease in net cell growth, the TGI value or cytostatic activity reported is the concentration of the compound resulting in total growth inhibition and the LC50 value corresponds to the cytotoxic activity and it is the concentration of the compound causing 50% loss of initial cells at the end of the incubation period (15). 

## Results and Discussion

In this study, seventeen derivatives from methyl (M1-M3), ethyl (E1-E8) and n-propyl (P1-P3) quinoxaline-7-caboxylate 1ˏ4-di-*N*-oxide and three di-reduced quinoxaline (DR1-DR3) derivatives (Table 1) were selected for *in-vitro* evaluation on 60 human tumor cell lines. Results are reported in Table 2. The number reported is growth relative to the no-drug control cells and it allows detection of both growth inhibition (values between 0-100) and lethality or cytotoxic activity (negative numbers). Compounds which reduce the growth of anyone of the cancer cell lines to 32% or values less are identified as “active” compounds (19).

**Table 1:** Quinoxaline-7-carboxylate 1,4-di-*N*-oxide derivatives selected by the National Cancer Institute for *in-vitro* evaluation on 60 human tumor cell lines

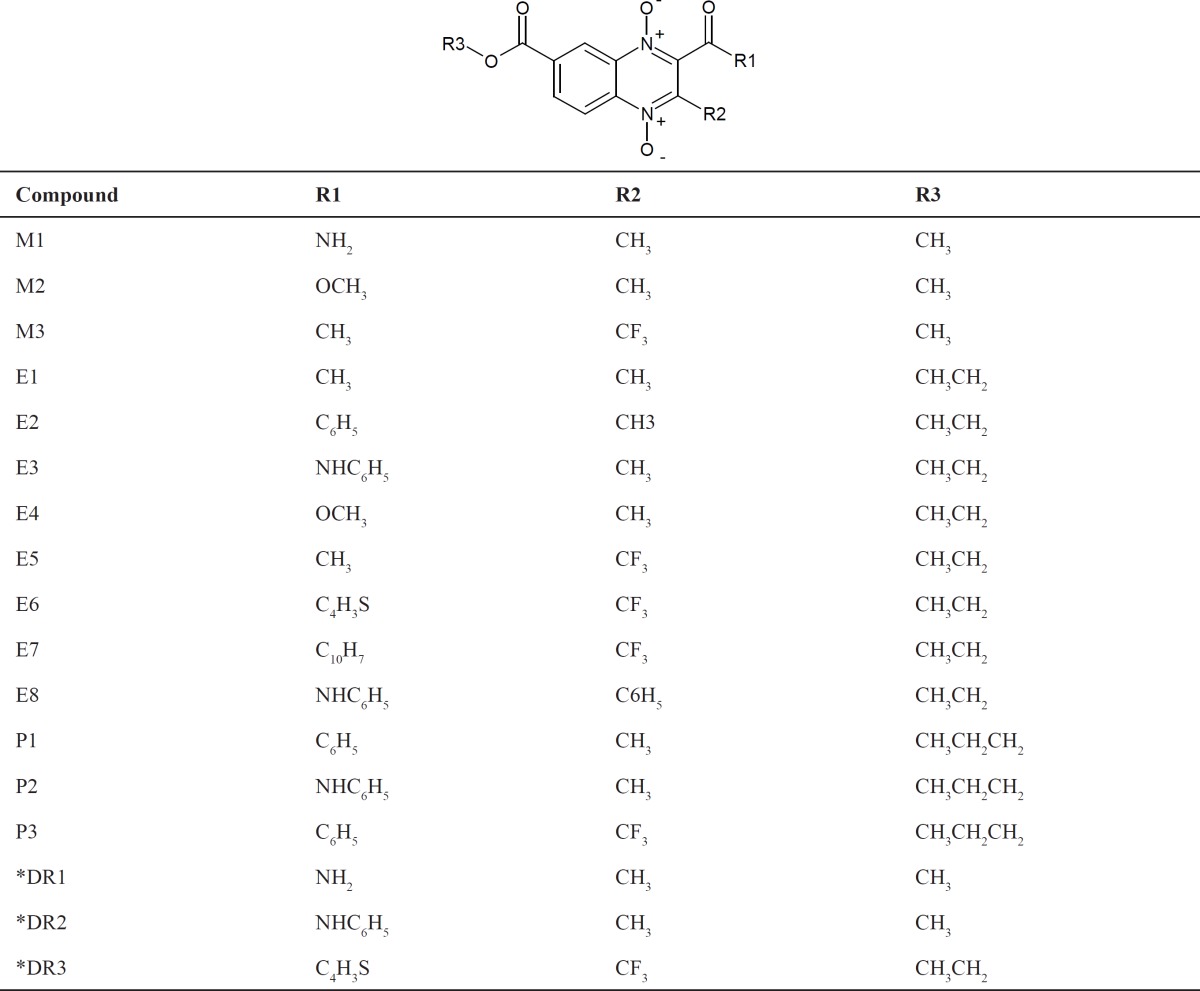

* Compounds DR1, DR2 and DR3, are di-reduced *N*-oxide derivatives.

**Table 2:** Cell growth inhibitory activity of ester of quinoxaline 1, 4-di-*N*-oxide at single dose assay (10^-5^ M).

**Panel/Cell Line**	**M3**	**E5**	**E6**	**E7**	**P3**
Leukemia					
CCRF-CEM	-	-18.11	-44.04	-	-64.45
HL-60(TB)	-	-65.92	-69.01	-	-71.61
K-562	-	-76.68	-52.64	-	-73.97
MOLT-4	-	-56.44	-67.27	-	-63.33
RPMI-8226	-48.00	8.56	-45.28	-66.78	-
SR	-	-59.23	-42.78	-	-41.36
Non-Small Cell Lung Cancer					
A549/ATCC	28.65	19.21	-94.87	-26.42	-88.10
EKVX	-46.30	29.13	-76.99	-56.85	-
HOP-62	-9.08	25.51	-90.62	-93.79	-96.53
HOP-92	-57.01	-29.91	-83.55	-	-
NCI-H226	-17.14	41.18	-54.87	-93.70	-
NCI-H23	-62.22	-16.94	-87.04	-88.42	-75.58
NCI-H322M	66.19	94.36	-87.64	67.25	-91.67
NCI-H460	-88.77	-50.66	-73.66	-100.0	-69.92
NCI-H522	-80.74	-83.76	-83.29	-80.11	-92.62
Colon Cancer					
COLO 205	-100.0	-78.26	-84.79	-100.0	-92.30
HCC-2998	-86.89	-17.11	-88.97	-97.59	-87.59
HCT-116	-97.97	-38.94	-96.22	-100.0	-73.00
HCT-15	-77.54	-35.95	-72.03	-96.09	-65.74
HT29	-56.03	-0.88	-82.43	-97.43	-92.15
KM12	-93.54	-85.51	-90.43	-91.99	-81.61
SW-620	-18.07	-0.80	-82.37	-89.55	-82.53
CNS Cancer					
SF-268	-24.84	-51.82	-89.64	-88.82	-81.45
SF-295	-45.72	23.01	-88.04	-84.83	-
SF-539	-45.85	29.71	-83.81	-100.0	-95.02
SNB-19	-6.44	17.38	-77.39	-85.51	-88.91
SNB-75	-20.53	25.58	-100.0	-100.0	-98.51
U251	-72.25	6.14	-100.0	-100.0	-
Melanoma					
LOX IMVI	-95.27	-59.67	-	-97.52	-74.66
MALME-3M	-26.39	-39.95	-	-69.07	-73.71
M14	-25.53	8.64	-96.58	-100.0	-70.27
MDA-MB-435	0.46	2.85	-80.51	-78.00	-89.53
SK-MEL-2	-31.89	12.15	-87.29	-83.53	-87.58
SK-MEL-28	26.96	27.93	-93.63	-96.34	-96.78
SK-MEL-5	-100.0	-94.33	-96.90	-100.0	-
UACC-257	-30.25	-37.08	-90.39	-71.67	-94.25
UACC-62	-32.68	2.99	-80.42	-97.18	-92.36
Ovarian cancer					
IGROV1	-78.34	-81.07	-60.32	-82.94	-74.39
OVCAR-3	-92.82	-84.74	-96.63	-99.57	-95.33
OVCAR-4	-41.96	-5.96	-83.35	-65.84	-73.09
OVCAR-5	-12.93	-1.19	-79.81	-98.53	-87.75
OVCAR-8	9.07	-42.56	-36.42	3.25	-85.96
NCI/ADR-RES	-40.71	6.85	-70.41	-86.40	-43.06
SK-OV-3	-31.41	24.84	-97.55	-100.0	-99.91
Renal cancer					
786-0	-40.52	11.40	-92.27	-100.0	-81.24
A498	6.79	12.45	-	-62.39	-90.87
ACHN	-99.32	5.29	-86.94	-100.0	-91.76
CAKI-1	-78.49	40.03	-85.42	-93.78	-92.47
RXF 393	-20.14	31.61	-92.28	-71.20	-93.23
SN 12C	-33.99	4.58	-47.28	-99.16	-92.33
TK-10	25.95	40.92	-96.50	-89.68	-99.13
UO-31	-81.97	27.45	-92.40	-96.00	-89.00
Prostate cancer					
PC-3	-58.10	-45.68	-75.44	-99.18	-91.49
DU-145	1.28	-88.45	-96.54	-100.0	-97.17
Breast Cancer					
MCF7	-50.33	-6.41	-68.11	-93.12	-69.69
MDA-MB-231/ATCC	-4.69	22.16	-76.77	-99.39	-90.36
HS 578T	-19.36	-23.86	-10.78	-45.74	-52.01
BT-549	-19.57	-44.86	-93.36	-48.46	-83.94
T-47D	-73.64	-26.07	-70.45	-91.10	-83.21
MDA-MB-468	-83.72	-37.04	-80.51	-92.65	-81.36
Mean	-41.01	-14.73	-79.03	-82.89	-82.75
Delta	58.99	79.60	20.97	17.11	17.16
Range	166.19	188.69	89.22	167.25	58.55

**Table 3:** Log10 GI50, Log 10 TGI, and Log 10 LC50 of the *in vitro* inhibitory activity test for compounds M-3, E-5, E-6, E-7 and P-3 against 60 human tumor cells lines

	**M-3**			**E-5**			**E-6**			**E-7**			**P-3**		
Panel/Cell Line	Log _10_ GI50	Log _10_ TGI	Log _10_ LC50	Log _10_ GI50	Log _10_ TGI	Log _10_ LC50	Log _10_ GI50	Log _10_ TGI	Log _10_ LC50	Log _10_ GI50	Log _10_ TGI	Log _10_ LC50	Log _10_ GI50	Log _10_ TGI	Log _10_ LC50
Leukemia															
CCRF-CEM	-5.34	-4.0	> -4.0	-5.73	-5.27	-4.25	**-6.67**	**-6.32**	-5.86	**-6.40**	-5.64	> -4.0	-5.73	-5.40	-5.07
HL-60(TB)	-5.71	-5.10	> -4.0	-5.73	-5.42	-5.11	**-6.56**	**-6.07**	-5.25	**-6.01**	-5.55	-5.09	-5.71	-5.41	-5.11
K-562	-5.64	-5.11	> -4.0	-5.97	-5.60	-5.23	**-6.72**	**-6.41**	**-6.10**	**-6.51**	-5.82	-5.23	-5.68	-5.39	-5.09
MOLT-4	-5.53	-4.75	> -4.0	-5.81	-5.23	-4.18	**-6.62**	**-6.28**	-5.54	-5.93	-5.48	-	-5.69	-5.39	-5.09
RPMI-8226	-5.65	-5.20	> -4.0	-5.78	-5.30	-4.17	-5.77	-5.35	-4.26	-5.96	-5.44	> -4.0	-5.71	-5.39	-5.06
SR	-5.64	-5.10	> -4.0	-	-	-	**-6.68**	**-6.38**	**-6.07**	**-6.48**	-5.80	> -4.0	-5.72	-5.31	-4.0
Non-Small Cell Lung Cancer															
A549/ATCC	-5.71	-5.43	-5.15	-5.60	5.24	-4.0	**-6.74**	**-6.44**	**-6.14**	-5.72	-5.44	-5.16	-5.78	-5.48	-5.18
EKVX	-5.71	-5.36	-5.01	-5.75	-5.40	-5.05	-5.89	-5.56	-5.22	-5.81	-5.50	-5.20	-	-	-
HOP-62	-5.71	-5.43	-5.15	-5.82	-5.48	-5.13	**-6.16**	-5.70	-5.35	-5.78	-5.51	-5.24	-5.75	-5.47	-5.20
HOP-92	-5.83	-5.50	-5.18	-5.84	-5.55	-5.26	**-6.77**	**-6.05**	-5.46	-5.92	-5.60	-5.28	-5.95	-5.62	-5.28
NCI-H226	-5.71	-5.44	-5.16	-5.74	-5.37	-4.98	**-6.16**	-5.60	-5.14	-5.76	-5.45	-5.13	-5.69	-5.33	-4.0
NCI-H23	-5.74	-5.47	-5.20	-5.84	-5.48	-5.11	**-6.01**	-5.64	-5.28	**-6.30**	-5.75	-5.37	-5.75	-5.47	-5.19
NCI-H322M	-5.37	-4.86	-4.36	-5.62	-5.17	-4.58	-5.80	-5.52	-5.24	-5.79	-5.52	-5.26	-5.74	-5.48	-5.23
NCI-H460	-5.74	-5.46	-5.18	-5.88	-5.57	-5.27	**-6.65**	**-6.25**	-5.64	-5.77	-5.49	-5.22	-5.69	-5.37	-5.05
NCI-H522	-5.78	-5.48	-5.19	-5.82	-5.44	-5.06	**-6.67**	**-6.23**	-5.61	**-6.34**	-5.73	-5.32	-5.74	-5.46	-5.17
Colon Cancer															
COLO 205	-5.71	-5.40	-5.10	-5.83	-5.53	-5.23	**-6.59**	**-6.22**	-5.67	-5.82	-5.54	-5.26	-5.68	-5.41	-5.15
HCC-2998	-5.75	-5.50	-5.24	-5.90	-5.57	-5.23	**-6.11**	-5.68	-5.31	-5.78	-5.52	-5.26	-5.74	-5.48	-5.22
HCT-116	-5.84	-5.56	-5.28	-5.92	-5.56	-5.20	**-6.77**	**-6.50**	**-6.23**	**-6.43**	-5.84	-5.42	-5.77	-5.47	-5.18
HCT-15	-5.74	-5.45	-5.17	-5.84	-5.44	-5.05	**-6.63**	**-6.20**	-5.59	-5.83	-5.48	-5.14	-5.79	-5.48	-5.18
HT29	-5.76	-5.49	-5.22	-5.79	-5.49	-5.18	**-6.77**	**-6.50**	**-6.22**	-5.97	-5.64	-5.32	-5.68	-5.39	-5.11
KM12	-5.75	-5.50	-5.24	-5.83	-5.52	-5.21	**-6.13**	-5.68	-5.31	-5.90	-5.60	-5.30	-5.74	-5.46	-5.18
SW-620	-5.71	-5.42	-5.12	-5.80	-5.42	-5.04	**-6.78**	**-6.47**	**-6.16**	-5.82	-5.54	-5.25	-5.73	-5.43	-5.14
CNS Cancer															
SF-268	-5.76	-5.49	-5.22	-5.84	-5.53	-5.21	**-6.73**	**-6.42**	-6.10	-5.94	-5.63	-5.31	-5.75	-5.4	-5.17
SF-295	-5.87	-5.56	-5.24	-5.87	-5.54	-5.21	-5.90	-5.59	-5.28	-5.75	-5.50	-5.25	-5.77	-5.49	-5.21
SF-539	-5.73	-5.47	-5.21	-5.79	-5.51	-5.23	-5.93	-5.62	-5.31	-5.81	-5.54	-5.27	-5.75	-5.49	-5.24
SNB-19	-5.51	-5.04	-4.52	-5.73	-5.39	-5.04	-5.94	-5.61	-5.29	-5.77	-5.51	-5.26	-5.76	-5.49	-5.23
SNB-75	-5.85	-5.57	-5.28	-5.81	-5.52	-5.24	-5.93	-5.62	-5.31	-5.95	-5.63	-5.32	-5.83	-5.52	-5.22
U251	-5.75	-5.49	-5.23	-5.81	-5.51	-5.21	**-6.79**	**-6.53**	**-6.26**	-5.82	-5.55	-5.27	-5.76	-5.49	-5.23
Melanoma															
LOX IMVI	-5.77	-5.49	-5.22	-5.85	-5.52	-5.20	**-6.77**	**-6.50**	**-6.24**	**-6.76**	**-6.48**	**-6.21**	-5.77	-5.47	-5.18
MALME-3M	-5.71	-5.42	-5.14	-5.81	-5.52	-5.22	**-6.50**	-5.84	-5.32	-5.77	-5.46	-5.15	-5.71	-5.44	-5.17
M14	-5.76	-5.49	-5.23	-5.79	-5.44	-5.08	**-6.18**	-5.70	-5.33	-5.84	-5.56	-5.28	-5.74	-5.47	-5.20
MDA-MB-435	-5.76	-5.44	-5.13	-5.82	-5.52	-5.22	**-6.61**	**-6.24**	-5.70	-5.82	-5.53	-5.24	-5.77	-5.49	-5.21
SK-MEL-2	-5.65	-5.32	-4.91	-5.69	-5.29	-4.48	-5.76	-5.48	-5.20	-5.74	-5.47	-5.20	-5.73	-5.45	-5.17
SK-MEL-28	-5.37	-4.88	-4.44	-5.44	-4.89	-4.43	-5.76	-5.51	-5.25	-5.78	-5.52	-5.26	-5.73	-5.45	-5.18
SK-MEL-5	-5.77	-5.51	-5.26	-5.84	-5.56	-5.27	-5.86	-5.57	-5.29	-5.84	-5.56	-5.27	-5.78	-5.51	-5.23
UACC-257	-5.74	-5.48	-5.22	-5.72	-5.42	-5.12	**-6.50**	-5.90	-5.45	-5.75	-5.49	-5.23	-5.76	-5.49	-5.23
UACC-62	-5.77	-5.50	-5.24	-5.86	-5.54	-5.23	-5.98	-5.64	-5.29	-5.82	-5.53	-5.25	-5.76	-5.49	-5.22
Ovarian Cancer															
IGROV1	-5.76	-5.47	-5.18	-5.80	-5.40	-5.0	**-6.72**	**-6.43**	**-6.15**	**-6.44**	-5.92	-5.44	-5.74	-5.44	-5.15
OVCAR-3	-5.74	-5.49	-5.23	-5.81	-5.51	-5.20	**-6.38**	-5.84	-5.40	**-6.13**	-5.69	-5.35	-5.76	-5.50	-5.24
OVCAR-4	-5.70	-5.36	-	-5.75	-5.41	-5.08	**-6.66**	**-6.27**	-5.60	**-6.58**	**-6.07**	-5.31	-5.76	-5.47	-5.19
OVCAR-5	-5.78	-5.51	-5.24	-5.81	-5.49	-5.16	-5.91	-5.60	-5.29	-5.89	-5.59	-5.30	-5.75	-5.48	-5.21
OVCAR-8	-5.54	-5.08	-4.34	-5.57	-4.91	-4.0	**-6.70**	**-6.38**	**-6.05**	-5.89	-5.57	-5.24	-5.74	-5.45	-5.16
NCI/ADR-RES	-5.61	-5.19	-4.10	-5.72	-5.15	-4.0	**-6.54**	**-6.06**	-5.31	-5.79	-5.48	-5.17	-5.72	-5.43	-5.14
SK-OV-3	-5.65	-5.35	-5.04	-5.79	-5.49	-5.20	-5.86	-5.58	-5.29	-5.79	-5.52	-5.26	-5.72	-5.48	-5.23
Renal Cancer															
786-0	-5.72	-5.47	-5.23	-5.80	-5.52	-5.23	**-6.27**	-5.73	-5.32	-5.79	-5.52	-5.26	-5.74	-5.48	-5.22
A498	-5.76	-5.48	-5.20	-5.87	-5.43	-4.96	-5.79	-5.51	-5.22	-5.78	-5.51	-5.24	-5.89	-5.55	-5.21
ACHN	-5.75	-5.49	-5.23	-5.80	-5.52	-5.23	**-6.77**	**-6.51**	**-6.26**	-5.83	-5.53	-5.23	-5.75	-5.49	-5.22
CAKI-1	-5.71	-5.29	-4.54	-5.84	-5.51	-5.18	**-6.67**	**-6.24**	-5.62	-5.85	-5.54	-5.24	-5.75	-5.47	-5.19
RXF 393	-5.69	-5.41	-5.14	-5.59	-5.06	-4.19	**-6.02**	-5.66	-5.32	-5.76	-5.49	-5.21	-5.77	-5.49	-5.21
SN 12C	-5.62	-5.31	-	-5.73	-5.42	-5.10	**-6.34**	-5.72	-5.21	-5.79	-5.45	-5.10	-5.75	-5.47	-5.20
TK-10	-5.56	-5.22	-4.71	-5.78	-5.41	-5.03	**-6.75**	**-6.47**	**-6.19**	-5.72	-5.48	-5.24	-5.80	-5.52	-5.25
UO-31	-5.77	-5.50	-5.24	-5.83	-5.50	-5.18	**-6.82**	**-6.54**	**-6.25**	-5.89	-5.59	-5.30	-5.78	-5.51	-5.25
Prostate Cancer															
PC-3	-5.78	-5.44	-5.10	-5.93	-5.57	-5.22	**-6.44**	-5.82	-5.30	**-6.14**	-5.70	-5.34	-5.81	-5.53	-5.25
DU-145	-5.74	-5.49	-5.24	-5.81	-5.52	-5.24	**-6.06**	-5.68	-5.34	-5.75	-5.50	-5.25	-5.73	-5.48	-5.23
Breast Cancer															
MCF7	-5.76	-5.47	-5.19	-5.93	-5.60	-5.26	**-6.44**	-5.88	5.32	**-6.05**	-5.62	-5.22	-5.79	-5.48	-5.17
MDA-MB-231/ATCC	-5.70	-5.41	-5.12	-5.76	-5.45	-5.13	**-6.40**	-5.81	-5.37	-5.80	-5.50	-5.20	-5.72	-5.46	-5.20
HS 578T	-5.63	-5.23	-4.0	-5.84	-5.35	-4.20	-5.67	-5.20	-4.0	-5.80	-5.44	-5.08	-5.70	-5.34	-4.00
BT-549	-5.68	-5.35	5.01	-5.84	-5.53	-5.22	**-6.31**	-5.75	-5.36	-5.81	-5.48	-5.15	-5.75	-5.49	-5.22
T-47D	-5.69	-5.38	-5.07	-5.85	-5.48	-5.11	**-6.31**	-5.73	-5.28	**-6.07**	-5.64	-5.25	-5.74	-5.43	-5.12
MDA-MB-468	-5.81	-5.52	-5.23	-5.80	-5.48	-5.17	**-6.51**	-5.78	-5.29	**-6.43**	-5.82	-5.39	-5.77	-5.45	-5.14

In the development of new anticancer drugs, tirapazamine, a quinoxaline 1,4-di-*N*-oxide derivative, is a potent antitumor agent, with a simple structure (small molecule) which induce a strong toxicity against a variety of tumor human cell (20). Therefore, our research group design and obtained new ester of quinoxaline 1, 4-di-*N*-oxide as potential antitumor agent. As shown in table 1, when analysing the compounds at single dose assay, compounds M3, E5, E6, E7 and P3 showed the highest global activity against the 60 human tumor cell lines tested. In all cases these compounds showed a negative final mean indicating cytotoxicity activity at 10^-5 ^M (-41.01, -14.73, -79.03, -82.89 and -82.75 respectively). In contrast to the other active compounds mentioned above, E5 do not show lethality against any of the renal cancer cells (values ranging 4.58 to 40.92). All other compounds showed little or no activity. None active compound showed selectivity to certain tumor cell type. As M3, E5, E6, E7 and P3 showed the highest anticancer activity, they were selected and assayed at five dose level against the same 60 human tumor cell lines panel. Results (Table 3) showed that compounds tested showed an excellent antitumor activity. E6 a quinoxaline 1ˏ4-di-*N*-oxide with ethyl ester at 7-position, was the most active with the lowest GI50, TGI and LC50 values against all the cell lines tested regardless of the tumour cell type, showing no selectivity. E7 showed its higher activity against leukemia and ovarian panel, while M3 showed good activity against the different tumour cell types except for leukemia with Log10 LC50 values >-4.0 M.Structure-activity relationship (SAR) analysis of methyl quinoxaline-7-carboxylate 1ˏ4-di-*N*-oxide (M1-M3) series shows that substitution at 3-position with electronegative group (CF_3_) play a very important role in the cytotoxicity activity, enhancing drastically biological effect. The same biological behaviour can be detected in ethyl (E1-E7) and n-propyl (P1-P3) quinoxaline-7-carboxylate 1ˏ4-di-*N*-oxide series, because only compounds substituted at 3-position with CF_3_ group showed inhibitory effect. Furthermore, the importance in the cytotoxic activity of CF_3_ group at 3-position on quinoxaline ring has been showed in the design and obtention of potent antitumor agents by Zarranz and cols (6). Substitutions at 2-position on quinoxaline 1,4-di-*N*-oxide ring increase the activity in the following order: naphthyl > thiophenyl > methyl. These results showed that aromatic and coplanar groups at 2-position are very important to enhance biological activity. This same key biological behaviour has been reported by other authors (6, 10). Finally, analysing compounds M3 and E5 shows that substitutions at 7-position with esters group not necessarily affect the cytotoxic activity, but the nature of this esters group could exert a strong influence on the selectivity. However, Ismail and cols mentioned that incorporation of halogen atom at 6 or 7-position could enhance activity, therefore, this is a point to consider in the near future to development new antitumor agents (12). Additionally results of compounds DR1-DR3 showed a great role of *N*-oxide group in the cytotoxic effects as previously has been reported (10, 12).

## Conclusion

In the light of these results and in order to further explore for this class of compounds, in the present study we reported the cytotoxic activities of ester of quinoxaline 1ˏ4-di-*N*-oxide derivatives selected by the NCI. The antitumor activity of all these compounds was evaluated against the NCI-60 cell line panel at a single dose assay (10^-5^ M) according to their own protocols. Five of the tested compounds were active ranging their activity from cytostatic to cytotoxic agents depending of the cell line used. Among the examined series, we found that compounds E7, P3 and E6 were the most active against all 60 human cell lines with global mean values of -82.89, -82.75 and -79.03 respectively. None compound showed selectivity to certain cancer cell type or to a particular cell line except E5, that was also considered active (-14.73) but do not show lethality against any of the renal cancer cells. The results of this investigation prompt us to continue the development and testing of novel quinoxaline 1ˏ4-di-*N*-oxide derivatives and importantly to carry out further studies to investigate their potential mechanisms of action.
